# False Impressions

**Published:** 1888-10-20

**Authors:** Caroline Fothergill

**Affiliations:** Author of "An Enthusiast," "A Voice in the Wilderness," etc.


### False Impressions.

By Caroline Fothergill, Author of "An Enthusiast," "A Voice in the Wilderness," etc.

(Continued from p. 31.)

### Chapter III.—Coming Home.

Beatrice's interview with Dr. Temple had taken place at the end of the winter, and a few days later she left Clearwater for Mayfield, which place she intended for the future to make her home. She had hitherto lived in the country, chiefly occupied in nursing her father, and with little scope for her somewhat original philanthropic schemes. After his death she decided to leave Blackmoor and establish herself in Mayfield, where she would find abundant occupation of the kind she loved. This very decision had proved Laura's helplessness, for she was altogether opposed to the idea, and did her best, though in vain, to dissuade Beatrice from carrying it out.

The occasion of her visit to the doctor, we know. She had made up her mind in silence, and had then announced her intention and proceeded to carry it out at once, again against Mrs. Ledward's judgment, and somewhat imperiously putting aside that lady's offer to accompany her. The expedition had ended ignominiously for her, and she could only console herself with the reflection that Dr. Temple lived at Clearwater, and she did not, so that it was in the highest degree improbable that she would ever see him again. The thought of the dark, cynical-looking man who had shared her waiting, disturbed her more than she cared to confess. He remained in her recollection in a way which she could not explain. She kept imagining him to have divined the ridiculous reason for which she had been there, and she felt sure that did he know it he would be unsparing in his mockery of such weakness. Here again, however, the chances were a thousand to one that she would never see him again. He probably lived at Clearwater, or if he did not she was scarcely likely, she told herself, to meet him at Mayfield.

She was not a perfect stranger at Mayfield, she had already some friends in the town, and her reputation as a beautiful, rich, and philanthropic woman had preceded her. People called at once, and she had in a very short time a large and growing circle of acquaintance. She received, too, reports and circulars from almost every charitable and philanthropic organisation in the town, with personal appeals for help and sympathy. She gave money in her lavish, careless way. She had an odd contempt for mere money-giving, especially when it cost neither effort nor sacrifice on the part of the donor; but she held aloof from active work with the various "causes" presented to her, and allied herself with only one society. The aim they proposed was new to her, she felt interested in it, and it harmonised with her own plans.

A band of enlightened and eminently practical men and women had formed themselves into a committee for the purpose of introducing a knowledge and love of pictures and all other beautiful things among the working people of Mayfield. These people, sufficiently numerous of themselves to form a fair-sized town, lived under most depressing and uncivilising conditions. Everyone knows what the "working quarter" of a large manufacturing town is like, and the working quarter of Mayfield combined all the bad features of other similar places, with some special ones of its own, an abnormally-high death-rate being one. Men and women living in these narrow, dreary streets, and children growing up in them, could not possibly have any conception of what a truly beautiful object is, or what an important and necessary part in our life truly beautiful things play. As far as art was concerned these people were dead, or at least sleeping, a deep, dreamless sleep, undisturbed by any waking hours in which they realised all there was in the world, of which they had no conception, but which might be brought within their reach. It was to bring art, with all its civ lising and ennobling influences, within reach of these people that this committee had formed itself into what was known as the "Murray Society." They did their work in a way which had probably never been tried before. They had bought in the heart of the working part of the town an old eighteenth-century house, which stood in a commanding position at the end of a black and dingy street, and in its own garden. Its ornamental brickwork and gabled façade seemed to separate it entirely from the hideous buildings—rows of dingy, monotonous cottages and railway sheds—with which it was surrounded, and marked it out as a conspicuous object—a bit of old-fashioned individualism in the midst of a wilderness of dreary uniformity. This was the "Murray Museum," and it contained a collection in many respects unique for completeness and perfection of arrangement, of pictures, photographs, coins, sculpture, pottery, fabrics, and art treasures of every description. The conduct of the museum, too, was unique. Different members of the committee attended during the afternoons and evenings (it was not open in the mornings), and put themselves at the service of the visitors, accompanying them through the rooms and galleries, explaining the different things, telling them about pictures, the different methods of producing pictures, what to look for in examining pictures, and so forth. There was a very interesting portrait gallery (in engravings), in which nearly every great artist, poet, literary or scientific man and woman was represented, and these engravings formed the subject of exceedingly interesting talks and discussions. Besides this, there were concerts, lectures, readings to children, classes for drawing, wood-carving and modelling, all free of charge, all admirably conducted, and highly appreciated; for the working people of Mayfield had justified the belief of the Murray Society, they had proved by their attendance at the museum and their interest in all they heard and saw there, that they had in reality been only sleeping, that when they were roused they could respond to the call.

The committee heard of Beatrice. One of the principal members was her partner at a dinner table, and entertained her the greater part of the time with an enthusiastic account of the museum and the good work it was doing. She was carried away by this novel form of philanthropy. The originality and reasonableness of the scheme appealed at once to her imagination. She paid a visit to the place under the guidance of her enthusiastic friend, and when, a little later, she received an invitation to join the committee, she readily accepted it. This was in the spring, but as comparatively little active work was done in the summer, and the committee met somewhat irregularly, and the hour of the meetings clashed with a temporary arrangement of her own, she was only present two or three times before the hot weather came, and she and Laura, like everyone else, left home for some weeks.

When she came back it was September, and the winter season was already beginning. She found thousands of things to do and invitations coming in in shoals. She and Mrs. Ledward had made a good impression upon Mayfield, Laura especially with her bright manner, and Beatrice was as popular as her somewhat peculiar disposition allowed. Her name and wealth of course caused her to take a somewhat prominent place.

Amongst these invitations was the one to Mrs. Lennox's garden party, which, as we know, was accepted, and where Beatrice and George met, and for a long time afterwards the recollection of that meeting disturbed her more than she would have confessed to anyone. She felt sure that this man had heard what she had said, and he could have only one opinion about it. The thought had flashed into her mind at once—

"He thinks I have been telling a lie."

It was this thought which constantly galled her later. If she prided herself upon one thing above another, it was upon her strict observance of and respect for the most delicate shades of truth. To her untruthfulness was the sin for which there is no forgiveness, and now she had been caught apparently tripping by someone to whom it was impossible she should ever justify herself. It mattered not that he was a stranger, nay, it made it so much the worse. The recollection of that little scene often darted into her mind when Laura was laughing at her for some manifestation of moral rigidity, and stung her pride sharply.

At the time of the garden party George Murray, like Beatrice, had only just returned to Mayfield, after an absence in his case of many months. He had left Clearwater the day after he had seen Beatrice there, and had spent his time since then in America, investigating social conditions and charitable organisations, and generally storing his mind with information. It was an engrossing occupation, and he had long intended to undertake it when he had the time, and he had found it even more interesting than he had expected. Nothing but the knowledge that he was really wanted at Mayfield would have induced him to come home again and put himself into the old harness.

He found the old house in Queen Street, which had been his home ever since he could remember, as smoke-begrimed and dull as ever, and this time he was very conscious that home-coming may be a dismal proceeding. The murky Mayfield sky seemed doubly depressing after the radiant heaven on the other side of the water, and his sister, Mrs. Lester, left something to be desired as a companion for life. She had welcomed him with all the affection of which she was capable, no doubt, but she had not an affectionate nature, and the assumption of it sat somewhat oddly upon her. She questioned him as to what he had seen and done, but they had never had much in common, and he did not feel inclined to tell her anything really intimate. Still, to do her justice, he had to confess that she was more amiable to him than to anyone else, and she certainly kept his house admirably for him. As a bachelor he would not have known what to do without her.

Besides, there was a little change. He had exchanged a few letters with his sister during his absence, and he had been aware that he would find an additional companion on his return. This was a slim, pretty girl of seventeen, with wavy light brown hair and large grey eyes, a niece by marriage of his sister—the only daughter of her husband's younger brother—and whom she had summoned to keep her company in the solitude in which George's departure left her. The girl's name was Edith Lester, and she had already been in Queen Street for three or four months when George returned. She seemed very shy and timid, scarcely spoke even to her aunt, unless first addressed, called George "Mr. Murray," and seemed desperately frightened of him.

On the very evening he came home his sister sent Edith to her room at an early hour, and then began to tell him about the girl. She had scarcely known her before this visit, it not being Mrs. Lester's habit voluntarily to seek out poor relatives, and she had been agreeably surprised by everything in her niece. Her humility, gratitude, and docility had caused her to become really fond of the girl, and she said she would like to adopt her and keep her as a permanent companion. She added that the arrangement would also be of immense benefit to Edith, who was naturally very delicate, and not fit to rough it in the home of a poor, overworked London clergyman, or to take the place which usually falls to a girl under those circumstances.

George made no difficulty about it at all. He said the house was large enough, another inhabitant would be an advantage, and Edith seemed very quiet; if his sister wished it, she might certainly stay. Her remaining possessions were therefore sent for, and she became a fixture in Queen Street.

It was only a week after his return that George went to Mrs. Lennox's party, the invitation to which he had found among many other similar things awaiting his home-coming. There to his extreme surprise, he saw Beatrice, and did actually carry on the conversation reported by Mrs. Ledward. He had left the house immediately after that, not trying to see Beatrice again. The feeling he had experienced on hearing her speak (he had only caught the latter half of her sentence, and so carried away a totally wrong impression) was of bewilderment, he also felt shocked, and a little disgusted. That any woman, especially a proud-looking woman like Miss Stanford, should condescend to falsehood, and for so trivial a reason, seemed to him contemptible beyond expression, and although he had not expressed himself exactly as Laura had reported, he was quite sincere in saying he did not wish to make Miss Stanford's acquaintance. There was a certain rigidity about him as about Beatrice, and this little incident caused his interest in Miss Stanford to considerably abate, if not altogether vanish. That same night he wrote the following letter to Dr. Temple :—

"My dear Temple,—

"Do you remember the case of Miss Stanford last February? Call it to mind if you can; also the conversation we had afterwards about her, your own panegyric on the lady, and my promise to let you know if I ever saw her again? I have seen her again this very day at a garden party, where I suddenly came upon Miss Stanford, very becomingly dressed, and looking exceedingly handsome, in the act of uttering, with perfectly unruffled countenance, a plain and unvarnished untruth. She was informing someone with charming candour, and how much veracity you and I know, that she had not consulted a doctor since she was a child. Immediately afterwards she saw and recognised me, and had the grace to look confused. But I only "turned on my heel" and walked away, nor did I see her again. Now what do you think of that? In our estimate of that woman, were you right, with your ideal theories and transendental notions, or was I when I said all women were by nature cowards? This little occurence has left a bad taste in my mouth, and my interest in Miss Stanford is gone. I care not if I never see her again.

"Truly yours,

"George Murray."

(*To be continued.*)

